# Audit of* Helicobacter pylori* Testing in Microbiology Laboratories in England: To Inform Compliance with NICE Guidance and the Feasibility of Routine Antimicrobial Resistance Surveillance

**DOI:** 10.1155/2016/8540904

**Published:** 2016-10-18

**Authors:** Rosalie Allison, Donna M. Lecky, Megan Bull, Kim Turner, Gauri Godbole, Cliodna A. M. McNulty

**Affiliations:** ^1^Primary Care Unit, Public Health England, Microbiology Department, Gloucestershire Royal Hospital, Great Western Road, Gloucester GL1 3NN, UK; ^2^Histology Department, Cheltenham General Hospital, Sandford Rd., Cheltenham, Gloucestershire GL53 7AN, UK; ^3^2gether NHS Foundation Trust, Rikenel, Montpellier, Gloucester GL1 1LY, UK; ^4^Bacteriology Reference Department, National Infection Service, Public Health England, 61 Colindale Avenue, London NW9 5EQ, UK

## Abstract

*Introduction*. The National Institute for Health and Clinical Excellence (NICE) guidance recommends that dyspeptic patients are tested for* Helicobacter pylori* using a urea breath test, stool antigen test, or serology. Antibiotic resistance in* H. pylori *is globally increasing, but treatment in England is rarely guided by susceptibility testing or surveillance.* Aims*. To determine compliance of microbiology laboratories in England with NICE guidance and whether laboratories perform culture and antibiotic susceptibility testing (AST).* Methods*. In 2015, 170 accredited English microbiology laboratories were surveyed, by email.* Results*. 121/170 (71%) laboratories responded; 96% provided* H. pylori* testing (78% on site). 94% provided* H. pylori *diagnosis using stool antigen; only four provided serology as their noninvasive test; 3/4 of these encouraged urea breath tests in their acute trusts. Only 22/94 (23%) of the laboratories performed* H. pylori* cultures from gastric biopsies on site; 9/22 performed AST, but the vast majority processed less than one specimen/week.* Conclusions*. Only five laboratories in England do not comply with NICE guidance; these will need the guidance reinforced. National surveillance needs to be implemented; culture-based AST would need to be centralised. Moving forward, detection of resistance in* H. pylori* from stool specimens using molecular methods (PCR) needs to be explored.

## 1. Introduction

*Helicobacter pylori* causes chronic active gastritis that may be associated with symptomatic dyspepsia, which occurs in over 10% of the UK adult population [1, 2]. Infection with* H. pylori* is a cofactor in the development of duodenal and gastric ulcers, gastric cancer, and gastric mucosa-associated lymphoid-tissue (MALT) lymphoma [3, 4]. Nevertheless, the great majority of patients with* H. pylori* infection will not have any clinically significant complications [5].

“Test-and-Treat” for* H. pylori* is recommended for symptomatic patients, as this strategy will cure most underlying peptic ulcer diseases and prevent future* H. pylori* associated gastroduodenal disease [2]. However, many infected patients with functional dyspepsia will not gain symptomatic benefit [6]. Current guidance from the National Institute for Health and Clinical Excellence (NICE) (Supplementary 1 in Supplementary Material available online at http://dx.doi.org/10.1155/2016/8540904) recommends that clinicians offer dyspeptic patients noninvasive* H. pylori* testing using a carbon-13 urea breath test (UBT) or a stool antigen test (SAT), or laboratory-based serology where its performance has been locally validated [2].

NICE recommends first-line treatment for* H. pylori* with a proton-pump inhibitor (PPI) and two antibiotics (usually amoxicillin and either clarithromycin or metronidazole), choosing the treatment regimen with the lowest acquisition cost, and taking into account previous exposure to clarithromycin or metronidazole [2].* H. pylori *treatment failure usually indicates poor compliance or antibiotic resistance, often acquired through antibiotic treatment for* H. pylori *or another infection [4]. A European prospective study (2008-2009) showed resistance rates of 17.5% for clarithromycin, 14.1% for levofloxacin, and 34.9% for metronidazole [7]. Routine surveillance of antibiotic resistance in* H. pylori* could inform treatment recommendations and assess the efficacy of public health strategies aiming to control antibiotic resistance.

This audit aimed to assess microbiology laboratory compliance with NICE guidance for the noninvasive diagnosis of* H. pylori* and to determine the number of laboratories undertaking culture and antibiotic susceptibility testing (AST). This data will be used to inform the need for enhanced implementation of the NICE guidance and decisions on how any future national* H. pylori* antibiotic resistance surveillance systems could be set up across England.

## 2. Methods

### 2.1. Questionnaire Development

The draft survey was developed by microbiologists with an interest in the field and piloted several times with five laboratories, for ease of completion and feasibility of data entry. The 14 survey questions (Supplementary 2) asked about the different diagnostic tests performed for* H. pylori* detection, including serology, stool antigen, culture from gastric biopsies, AST, PCR, rapid urease on biopsies and C^14^ breath tests, numbers performed weekly/yearly, and typical positivity rates.

### 2.2. Participants

Questionnaires were sent by email, in 2015, to 170 accredited microbiology laboratories in England. All nonresponding laboratories were telephoned to check the name and contact details of a suitable contact involved in* H. pylori* testing. Nonresponders could also complete the questionnaire by telephone. To increase returns, questionnaires were also circulated via Public Health England (PHE) laboratory circulation lists. Participants were offered a certificate to use towards their Continuing Professional Development (CPD) as an incentive to complete the questionnaire.

### 2.3. Data Analysis

EpiData version 3.1 was used to input the questionnaire data. Data was cleaned by one of the research team. Where laboratories had ticked more than one box for an answer, comments given by participants were used by the researchers to determine which answer was counted and resolve discordant results. Free text answers were discussed by three researchers and assigned to categories. The questionnaire data was double entered by two separate researchers; minor disagreements were resolved by referral to the original questionnaire. The data was exported to Microsoft Excel 2010 for data analysis. The number of responses and proportion of different responses to each question were tabulated and summary descriptive statistical analysis was carried out and graphs were produced to display the data.

## 3. Ethics

This audit was undertaken to assess the need for routine national surveillance for antimicrobial resistance and as such did not require ethical approval. However, the audit was undertaken in line with information governance.

## 4. Results and Discussion

**Figure 1:**
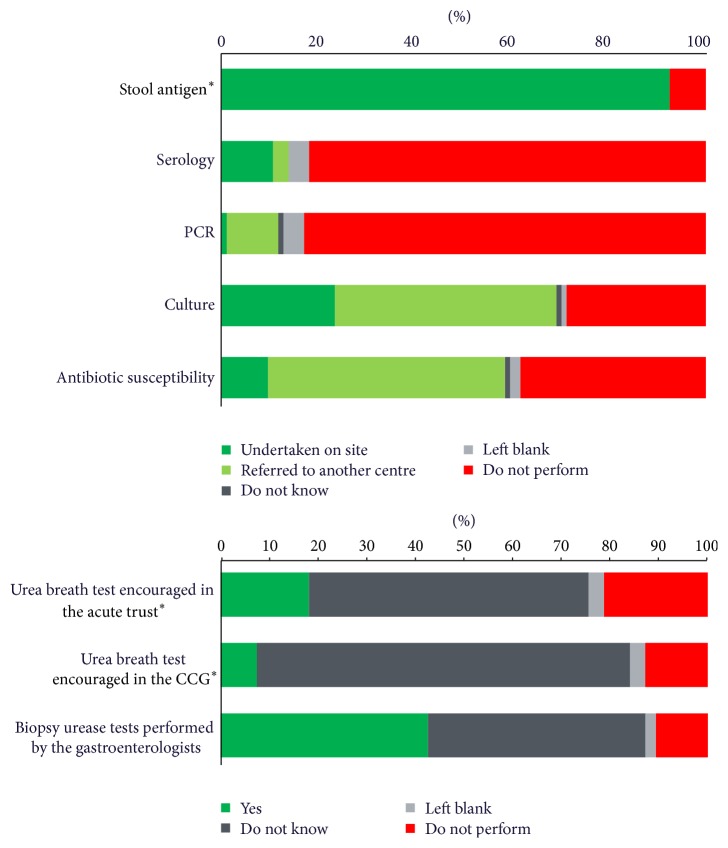
The number of laboratories offering various* Helicobacter pylori* diagnostic tests on site or through a referral service (*n* = 94). ^*∗*^Recommended by NICE.

Of the 121/170 (71%) laboratories that responded, 116/121 (96%) provided a* H. pylori* testing service: 94/121 (78%) of which were performed on site and 22/121 (18%) referred specimens to another laboratory (see Figure 1). Of the five laboratories not providing a testing service, two reported that their gastroenterologists performed urea breath tests.

### 4.1. Compliance with NICE Recommendations for the Diagnosis of* Helicobacter pylori*

**Figure 2:**
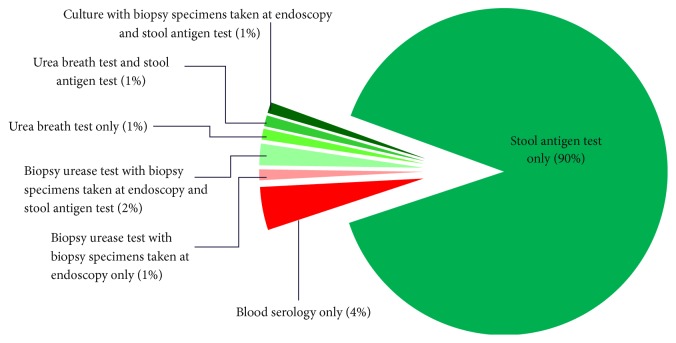
First-line diagnostic* Helicobacter pylori* tests performed on site by laboratories (*n* = 94).

Figure 2 shows that the results of this audit are promising, as a vast majority of laboratories (88/94, 94%) are providing stool antigen testing to diagnose* H. pylori*, which is the laboratory diagnostic test recommended in the NICE guidance [2]. However, there are still four laboratories doing serology as their first-line noninvasive diagnostic test, although three of these reported that they encourage urea breath tests in their acute trusts.

### 4.2. Stool Antigen Tests

**Figure 3:**
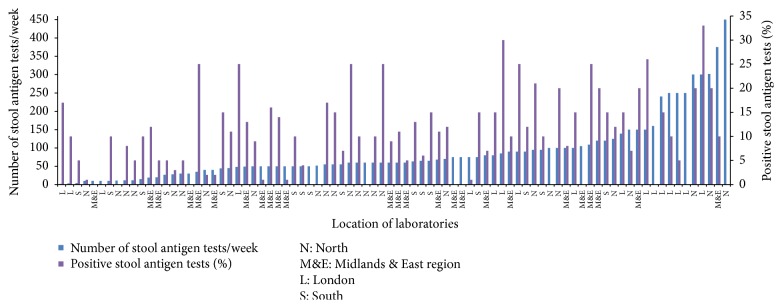
Average reported* H. pylori* stool antigen tests performed in-house each week with percentage positive results (*n* = 88).

The number of* H. pylori* stool antigen tests performed by each lab ranged from 2/week to 450/week, with a mean of 88 tests/week (see Figure 3). Individual laboratories reported positivity rates of between 1% and 33% (mean 13%). There is a very large range in the positivity rate for* H. pylori* stool antigen tests (Figure 3) indicating that different patient groups are being tested in the centres with high and low positivity. A high percentage of positive results could be because either clinicians are only doing the test after treatment failure or, alternatively, the population has high ethnicity or deprivation, in which prevalence is higher [4, 8–10]. To explore this, indices of multiple deprivation (IMD, 2015) of the Clinical Commissioning Groups (CCGs) served by each laboratory were examined, and no correlation was found between positivity of* H. pylori* diagnosis by stool antigen test and deprivation (*R* ^2^ = 0.0233). However, no data on ethnicity was provided.

In some laboratories, the positivity rate of their stool antigen tests was reported to be 3% or less (minimum of 1%), which is very low. False negative stool antigen or urea breath tests can be obtained if a patient is still taking proton-pump inhibitors or antibiotics when the test is performed, as they can reduce numbers of* H. pylori* in the gastric mucosa [11]. It is possible that, in these laboratories, patients were not advised to take or did not stop taking proton-pump inhibitors two weeks before, or antibiotics for 4 weeks before, the stool antigen test (as advised in NICE guidance) [2, 11]. However, you would expect noncompliance with this recommendation to be similar throughout the country. Therefore, it is likely that the varying positivity rate in labs represents either a true variation in the* H. pylori *rate across England, which would influence dyspepsia and ulcer rates, or a variation in testing guidance for clinicians [4]. This needs to be further investigated with more detailed epidemiological surveillance comparing patient demographics and details of the clinical indication for the test.

### 4.3. Serological Testing for* H. pylori*

Ten laboratories performed* H. pylori* blood serology, six of which were in addition to stool antigen testing. Three laboratories (3%) refer their blood serology to other laboratories, and 77 laboratories (82%) reported that they do not perform* H. pylori* blood serology. On average, 48 tests were performed in the laboratories each week and the percentage of positive* H. pylori* serology tests reported by 6 laboratories ranged from 5% to 25% with a mean of 13% positive. Three laboratories commented that blood serology was only used as second-line therapy by gastroenterologists for patients on* Helicobacter* suppressing drugs such as PPI or for patients that had an active bleeding ulcer. This is in line with National PHE guidance that advises that serology is useful in patients with acute gastrointestinal bleeding when blood and PPI use may interact with the UBT or SAT [12].

### 4.4. PCR

Of the 94 laboratories that routinely test for* H. pylori*, 1 laboratory (1%) performed* H. pylori* PCR tests in their lab, 10 laboratories (11%) referred samples to other laboratories (9 to the GBRU), and 78 laboratories (83%) do not offer this service.

### 4.5. Urea Breath Test

Urea breath test is the most reliable noninvasive test for* H. pylori* [13]. In the UK, unlike other diagnostic tests, it needs to be prescribed, as the test involves taking a capsule containing radiolabelled urea, 20 to 30 minutes before the breath test is performed, and then sent to a central laboratory for analysis. Most laboratories did not know whether urea breath tests (UBTs) were encouraged: in their hospital setting (57%) or CCG (77%). Only 17 (18%) laboratories reported that UBTs were encouraged within their acute trust and 7% reported that UBTs were encouraged by their CCGs. It could be advised that laboratory staff should discuss whether UBTs are encouraged by their gastroenterologists in the acute trust or community, as this could influence what tests the laboratory should be providing or advising, before and after* H. pylori* treatment.

### 4.6. Biopsy Urease and Culture of Biopsy Specimens

Almost half of the laboratories, 46% (43/94), referred biopsy specimens for culture to another laboratory: 39/43 (91%) to the Gastrointestinal Bacteria Reference Unit (GBRU) in London. 40 laboratories (43%) performed* H. pylori* biopsy urease tests.* H. pylori* cultures were performed on site by 22/94 laboratories. Three of the laboratories that performed cultures on site also referred some specimens of cultures to the Reference Unit.

Only two laboratories reported culturing a significant number of gastric biopsy specimens for* H. pylori *(10 or more gastric biopsies per week) and the other 20 performed it occasionally (processing less than one biopsy culture weekly). Seven laboratories commented that they rarely carried out cultures of biopsy specimens; and another eight reported that culture was only performed after treatment failure. The two laboratories that performed more than ten specimens reported their positivity as 7% and 30%.

### 4.7. Antibiotic Susceptibility

Of the 22 laboratories that performed in-house cultures of biopsy, nine reported testing for antibiotic susceptibility on site, and nine referred their samples (8/9 referred to the GBRU). Four referred the biopsy specimens, three referred culture isolates, and two referred both biopsy specimens and culture isolates.

Of the nine laboratories that test for antibiotic susceptibility in-house, only one tested all five antibiotics listed as first- or second-line therapy in NICE guidance (metronidazole, clarithromycin, amoxicillin, tetracycline, and levofloxacin) and only another one tested for levofloxacin [2] (see Table 1).

The most recent data shows that the current combination treatment has lost some efficacy with successful eradication rates in about 70% of patients [14].* H. pylori* treatment failure is often due to antibiotic resistance [3, 4, 15–17]. Despite this, a surveillance system to monitor trends in resistance at a local and national level does not currently exist in England. The tailored treatment regimen chosen for* H. pylori* could be based on culture and susceptibility testing of gastric biopsy specimens from each individual patient [14, 15, 18–20]. However, this is not cost-effective in a country with low prevalence, such as England. Thus, NICE guidance recommends Test-and-Treat for* H. pylori* using noninvasive tests for nonulcer dyspepsia [2, 21]. NICE guidance only recommends the invasive approach of culture and susceptibility testing, after treatment failure [2]. Despite this, antibiotic resistance surveillance is needed to inform the empirical treatment guidance used in an area for Test-and-Treat of patients with dyspepsia. Such an antibiotic resistance surveillance service is running in Germany [22].

This audit indicates that only two laboratories routinely cultured significant numbers of gastric biopsy specimens for* H. pylori*. It is important that these laboratories continue to be supported to provide this service in order to maintain their expertise and provide resilience. These laboratories could provide a regional service for biopsy culture-based antibiotic resistance surveillance. Alternatively, culture-based surveillance could be provided centrally by the Gastrointestinal Bacterial Reference Unit (GBRU).

Robust national surveillance is required to quantify the burden of disease and microbiological surveillance is necessary for monitoring antimicrobial resistance (AMR). Currently, the rates of AMR to first-line agents for empirical treatment of* H. pylori* in England are not known, and we do not know whether the threshold of resistance (20%) for changing the combination has been reached.

Although it is not current practice, we recommend that patient demographic data, including indication for the test, should be routinely collected. As most laboratories perform stool antigen tests, this demographic and clinical data could be used to monitor* H. pylori* prevalence rates in different sectors of the population. Furthermore, commercial assays for detection of AMR in* H. pylori* in the stool specimens are available and could prove to be a useful tool for rapid detection of resistance to first-line agents like macrolides and quinolones [23–25]. The use of multiple antibiotics for a prolonged period of time for the treatment of recurrent* H. pylori* is an important aspect of community based antimicrobial stewardship which needs to be addressed.

### 4.8. Strengths and Limitations

A strength of this audit is the very high (71%) questionnaire return from the microbiology department; it is possible that nonresponding laboratories do not test for* H. pylori* on site. A limitation is that the questionnaire was not sent to gastroenterologists or histopathologists, so it is not known which diagnostic services they provide.

As specific patient details are unknown, the laboratory reported test positivity rates cannot be verified, and it is unknown whether individuals were tested more than once within the given time period. For future audits, it could be recommended that duplicates are removed so that positivity rates can be more rigorously followed.

## 5. Implications

The majority of laboratories comply with NICE guidance by undertaking stool antigen tests. However, four laboratories still perform serology as their first-line diagnostic test. As very few laboratories are routinely performing culture of gastric biopsy specimens to test for AMR, an English culture-based surveillance system would probably need centralised culture. However, a PCR based stool specimen surveillance system could be possible. The feasibility of stool based surveillance for monitoring prevalence would be simple to set up with some extra clinical data collection. Further work is required to determine the feasibility, cost-effectiveness, sensitivity, and specificity of the stool based molecular assays for detection of AMR. However, such a service would be immensely helpful to monitor AMR to* H. pylori* in the community setting.

## Supplementary Material

Supplementary 1. Gastro-oesophageal reflux disease and dyspepsia in adults: investigation and management NICE Guidelines (CG184) (2014). The guidance documents recommended Helicobacter pylori testing and eradication methods.Supplementary 2. Questionnaire sent to laboratories requesting information on which tests are routinely performed to diagnose H. pylori and their current practice on testing for antibiotic susceptibility.

## Figures and Tables

**Table 1:** Antibiotic susceptibility tests performed in-house by laboratories (*n* = 8).

Agents tested	Number of labs (%)	Number of labs performing each test (can do both)	
		*e*-test	Disc.
Metronidazole	*n* = 7 (88%)	4	4
Clarithromycin	*n* = 6 (75%)	4	2
Amoxicillin	*n* = 7 (88%)	4	3
Tetracycline	*n* = 5 (63%)	4	1
Levofloxacin	*n* = 2 (25%)	1	1

## Abbreviations

GBRU: Gastrointestinal Bacterial Reference Unit
NICE: National Institute for Health and Clinical Excellence
AST: Antibiotic susceptibility testing
AMR: Antimicrobial resistance
PPI: Proton-pump inhibitor
MALT: Mucosa-associated lymphoid-tissue
PCR: Polymerase chain reaction
PHE: Public Health England
UBT: Urea breath test
SAT: Stool antigen test
CCG: Clinical Commissioning Group
CPD: Continuing Professional Development.
