# Supplementary material for: Audit of Helicobacter pylori Testing in Microbiology Laboratories in England: To Inform Compliance with NICE Guidance and the Feasibility of Routine Antimicrobial Resistance Surveillance
Source: Int J Microbiol. 2016 Oct 18;2016:8540904. doi: 10.1155/2016/8540904 (PMC5088324; doi:10.1155/2016/8540904)
Supplement: Supplementary file 1 — Supplementary 1. Gastro-oesophageal reflux disease and dyspepsia in adults: investigation and management NICE Guidelines (CG184) (2014). The guidance documents recommended Helicobacter pylori testing and eradication methods.Supplementary 2. Questionnaire sent to laboratories requesting information on which tests are routinely performed to diagnose H. pylori and their current practice on testing for antibiotic susceptibility. [file 8540904.f1.pdf]

Supplementary Material

Supplementary 1. NICE guidance (2014)

**Gastro-oesophageal reflux disease and dyspepsia in adults: investigation and management**<br>**NICE Guidelines (CG184)**<br>**Published date: September 2014**
**1.9 *Helicobacter* pylori testing and eradication**<br>**Testing**<br>**1.9.1.** Test for *H pylori* using a carbon-13 urea breath test or a stool antigen test, or laboratory-based serology where its performance has been locally validated. **[2004, amended 2014]**<br>**1.9.2.** Perform re-testing for *H pylori* using a carbon-13 urea breath test. (There is currently insufficient evidence to recommend the stool antigen test as a test of eradication.) **[2004]**<br>**1.9.3.** Do not use office-based serological tests for *H pylori* because of their inadequate performance. **[2004, amended 2014]**
**Eradication**<br>**First-line treatment**<br>**1.9.4.** Offer people who test positive for *H pylori* a 7-day, twice-daily course of treatment with:<br>a PPI **and** amoxicillin **and** either clarithromycin or metronidazole.

Supplementary 2. Questionnaire sent to laboratories requesting information on which tests are routinely performed to diagnose *H. pylori* and their current practice on testing for antibiotic susceptibility

LABORATORY SURVEY 2015
WHAT LABORATORY TESTS ARE PROVIDED FOR *HELICOBACTER PYLORI*?

NICE have recently released new guidance on the management of dyspepsia and will soon be releasing standards on its diagnosis and management; this may include the diagnosis of *Helicobacter pylori.* Currently there is no data on the types of tests undertaken and in which

microbiology labs these tests are performed for the diagnosis of *H. pylori* to inform these standards. We do hope you will therefore take just a few minutes to answer the following short questions.

1. Lab name: **2. Date:** ____/____/_______ (dd/mm/yyyy)

3. Name of respondent: **4. Email:**

5a. Population served …………………………… **Certificate for CPD?** ☐ Yes ☐ No
(will be emailed to address given above)

5b. Do you refer your H pylori samples elsewhere? If so, where ………………………………

5c. Other Laboratories served by you:

……………………………………………………………………….

6. Is *Helicobacter pylori* blood serology performed in your laboratory?

☐ Yes: please state make of test used ……………………………………………………..………

☐ No: If no, which lab do you refer your samples to ....

If yes, approximately how many per week? ……………………………………….……………..

If yes could you provide an approximate percentage that are positive..

Is this test perfomed on specific patient groups only? ☐ No ☐ Yes,

If yes which groups?..

Comments……………………………………………………………………………………………

7. Are *Helicobacter pylori* stool antigen tests performed in your laboratory?

☐ Yes, please state make of test used ……………………………………………………………

☐ No ☐ Don't know

If yes, approximately how many per week? ……………………………………………………

If yes could you tell me the approximate percentage that are positive? ………………….............

Comments………………………………………………………………………………………………

8. Is culture of biopsy specimens for *Helicobacter pylori* performed in your laboratory?

☐ Yes ☐ No ☐ Don't know

If yes, approximately how many per year? ……………………………………………………………

If yes could you tell me the approximate percentage that are positive? …………………

If culture is not performed **in- house** are biopsy specimens referred elsewhere for culture?

☐ Yes ☐ No ☐ Don't know

If yes, please specify where……………………………………………………..…

Are there specific clinical scenarios in which cultures are performed?

……………………………………………………………………………………………………...

9. Is antibiotic susceptibility testing for *Helicobacter pylori* performed in your trust?

☐ No ☐ Yes If yes, what agents are tested?

☐ metronidazole	☐ disc	☐ Etest
☐ clarithromycin	☐ disc	☐ Etest
☐ amoxicillin	☐ disc	☐ Etest
☐ tetracycline	☐ disc	☐ Etest
☐ levofloxacin	☐ disc	☐ Etest

☐ others specify………………………………………………………………………………………..

If susceptibility tests are not done in-house, are biopsy specimens or isolates referred elsewhere for this?

☐ Yes, biopsy specimens ☐ Yes, culture isolates ☐ No

If yes, specify to which laboratory: ………………………………………………………………………

10. Are any *Helicobacter pylori* PCR tests performed in your lab?

☐ Yes ☐ Don't know ☐ No ☐ No, referred elsewhere (to ……………………………..………lab)

If yes, please specify the tests………………………………………………………………………………

11. Do you know if *Helicobacter pylori* biopsy urease tests are performed by your gastroenterologists?

☐ Yes, ☐ No, ☐ Don't know

Comments ……………………………………………………………………………………………………

***Helicobacter pylori* breath tests are detailed in the BNF and are available to patients on prescription**

12. Do you know if *Helicobacter pylori* breath tests are encouraged in your acute trust?

☐ Yes ☐ No ☐ Don't know

13. Do you know if *Helicobacter pylori* breath tests are encouraged by your CCG?

☐ Yes ☐ No ☐ Don't know

14. What is your first line test for the diagnosis of *Helicobacter pylori*?

- ☐ Stool antigen test
- ☐ Blood serology
- ☐ Biopsy urease test with biopsy specimens taken at endoscopy
- ☐ Histopathology with biopsy specimens taken at endoscopy
- ☐ Urea breath tests
- ☐ Culture with biopsy specimens taken at endoscopy
- ☐ PCR with biopsy specimens taken at endoscopy
- ☐ We do not routinely test for *Helicobacter pylori*

Thank you for taking the time to answer these questions. Please return as soon as possible

by email to: rosie.allison@phe.gov.uk

By post to: **Primary Care Unit, Microbiology, Gloucestershire Royal Hospital, Great Western Road, Gloucester, GL1 3NN.** *If you have any queries please contact Dr Cliodna McNulty on 0300 422 5066*
